# Methanotrophic Flexibility of ‘*Ca*. Methanoperedens’ and Its Interactions With Sulphate‐Reducing Bacteria in the Sediment of Meromictic Lake Cadagno

**DOI:** 10.1111/1462-2920.70133

**Published:** 2025-07-10

**Authors:** Maider J. Echeveste Medrano, Guangyi Su, Lucas A. Blattner, Pedro Leão, Irene Sánchez‐Andrea, Mike S. M. Jetten, Cornelia U. Welte, Jakob Zopfi

**Affiliations:** ^1^ Department of Microbiology Radboud Institute for Biological and Environmental Sciences, Radboud University (RIBES) Nijmegen the Netherlands; ^2^ Department of Environmental Sciences University of Basel Basel Switzerland; ^3^ Laboratory of Microbiology Wageningen University and Research Wageningen the Netherlands; ^4^ Science and Technology School IE University Segovia Spain

## Abstract

The greenhouse gas methane is an important contributor to global warming, with freshwater sediments representing important potential methane sources. Anaerobic methane‐oxidising archaea mitigate methane release into the atmosphere by coupling the oxidation of methane to the reduction of extracellular electron acceptors or through interspecies electron transfer with microbial partners. Understanding their metabolic flexibility and microbial interactions is crucial to assess their role in global methane cycling. Here, we investigated anoxic sediments of the meromictic freshwater Lake Cadagno (Switzerland), where ‘*Ca*. Methanoperedens’ co‐occur with a specific sulphate‐reducing bacterium, with metagenomics and long‐term incubations. Incubations were performed with different electron acceptors, revealing that manganese oxides supported highest CH_4_ oxidation potential but enriched for ‘*Ca*. Methanoperedens’ phylotypes that were hardly present in the inoculum. Combining data from the inoculum and incubations, we obtained five ‘*Ca.* Methanoperedens’ genomes, each harbouring different extracellular electron transfer pathways. In a reconstructed *Desulfobacterota QYQD01* genome, we observed large multi‐heme cytochromes, type IV pili, and a putative loss of hydrogenases, suggesting facultative syntrophic interactions with ‘*Ca.* Methanoperedens’. This research deepens our understanding of the metabolic flexibility and potential interspecific interactions of ‘*Ca.* Methanoperedens’ in freshwater lakes.

## Graphical abstract

In anoxic sediments of the meromictic freshwater Lake Cadagno ‘*Ca*. Methanoperedens’ and sulphate‐reducing *Desulfobacterota QYQD01* co‐occur. Metagenomics revealed that ‘*Ca.* Methanoperedens’ genomes harbour different extracellular electron transfer pathways. In the reconstructed *Desulfobacterota QYQD01* genome, we observed large multi‐heme cytochromes, type IV pili, and a putative loss of hydrogenases, suggesting facultative syntrophic interactions with ‘*Ca.* Methanoperedens’.

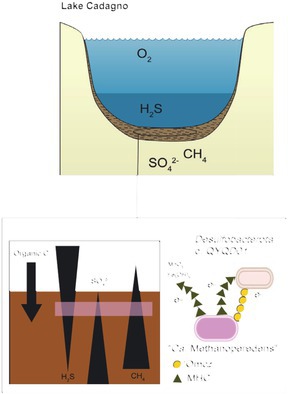

## 1 Introduction

Anaerobic oxidation of methane (AOM) is an important biological sink for this potent greenhouse gas (Knittel and Boetius 2009; Saunois et al. 2020; Wallenius et al. 2021) in a wide range of anoxic ecosystems, including inland waters, coastal and ocean ecosystem sediments (Rosentreter et al. 2021; Gao et al. 2022; Su et al. 2022). AOM is catalysed via reverse methanogenesis by different groups of anaerobic methane‐oxidising (ANME) archaea (Timmers et al. 2017; Chadwick et al. 2022) using a variety of electron acceptors (Glodowska et al. 2022). In marine sediments, sulphate dependent‐AOM (S‐AOM) is the predominant process, carried out by the ANME groups 1, 2A, 2B, 2C and 3 in syntropy with sulphate‐reducing bacteria (SRB) (Metcalfe et al. 2021; Yu et al. 2021; Murali et al. 2023). The ANMEs and SRBs engage in direct interspecies (DIET) or extracellular electron transfer (EET) via multi‐heme *c*‐type cytochromes (MHC) or conductive pili and/or other intermediates (Wegener et al. 2015; Scheller et al. 2016; Krukenberg et al. 2018). In freshwater systems, sulphate is much less abundant and the ANME‐2D group, ‘*Candidatus (Ca.)* Methanoperedens’ spp., appears to drive AOM with nitrate, humic acids, or metal oxides as electron acceptors without the need for a syntrophic partner (Haroon et al. 2013; Ettwig et al. 2016; Vaksmaa et al. 2017; Cai et al. 2018; Leu et al. 2020; Valenzuela et al. 2020; Cai et al. 2022; Pelsma et al. 2023). However, interactions with certain guilds such as nitrite‐scavenging anammox or *Methylomirabilis* bacteria might be beneficial for ‘*Ca*. Methanoperedens’ (Arshad et al. 2017; Dalcin Martins et al. 2022).

Reports of ‘*Ca*. Methanoperedens’ spp. in estuarine and marine habitats are rather scarce, although enrichment cultures have been shown to withstand marine salinities (Frank et al. 2023; Echeveste Medrano et al. 2024). Enrichment cultures of ‘*Ca*. Methanoperedens’ have usually been established from freshwater source material, with different electron acceptors including nitrate, manganese‐ and/or iron oxides. These studies revealed that the reduction of nitrate is performed by a membrane‐bound nitrate reductase within the respiratory chain, while large MHC‐containing proteins are predicted to engage in EET for insoluble metal oxides or electrodes (Leu et al. 2020; Cai et al. 2022; Zhang et al. 2023; Ouboter et al. 2024). As MHCs are also responsible for the electron transfer between S‐AOM ANMEs and their partner SRB bacteria (Meyerdierks et al. 2010; McGlynn et al. 2015), the presence of multiple MHCs in ‘*Ca*. Methanoperedens’ spp. begs the question of whether they could also live in syntropy with SRBs in sulphate‐rich freshwater ecosystems.

One of the earliest reports of the potential of ‘*Ca*. Methanoperedens’ spp. mediating freshwater S‐AOM came from sediments of the meromictic Lake Cadagno (Switzerland) (Schubert et al. 2011), where anoxic incubation experiments with methane, different electron acceptors, combined with molybdate as an inhibitor of bacterial sulphate reduction, revealed that AOM was predominantly sulphate‐dependent (Su et al. 2020). Moreover, 16S rRNA gene‐based analyses showed a striking co‐occurrence along the sediment depth profile of several ‘*Ca*. Methanoperedens’ Amplicon Sequencing Variants (ASVs) and one single ‘*Desulfobulbaceae*’ ASV, now reclassified as ‘uncultured *Desulfobacterota’* according to SILVA v138.2. This observation suggests that ‘*Ca*. Methanoperedens’ is probably not responsible for the reduction of sulphate itself but might oxidise methane in syntrophy with an SRB partner.

In this study, we aim to elucidate the diversity and metabolic potential of the ‘*Ca*. Methanoperedens’ phylotypes in the sediments of Lake Cadagno, along with that of the putative syntrophic SRB partner, previously inferred based on co‐occurrence patterns (Su et al. 2020). For this, we combined data from long‐term sediment incubations with different electron acceptors and ^13^C‐methane oxidation, 16S rRNA gene amplicon sequencing and metagenomic analyses. Our findings deepen the understanding of the mechanisms involved in freshwater S‐AOM, and support a potential syntrophic interaction with ‘*Ca*. Methanoperedens’ and a co‐occurring *Desulfobacterota*.

## 2 Materials and Methods

### 2.1 Study Workflow

**FIGURE 1:**
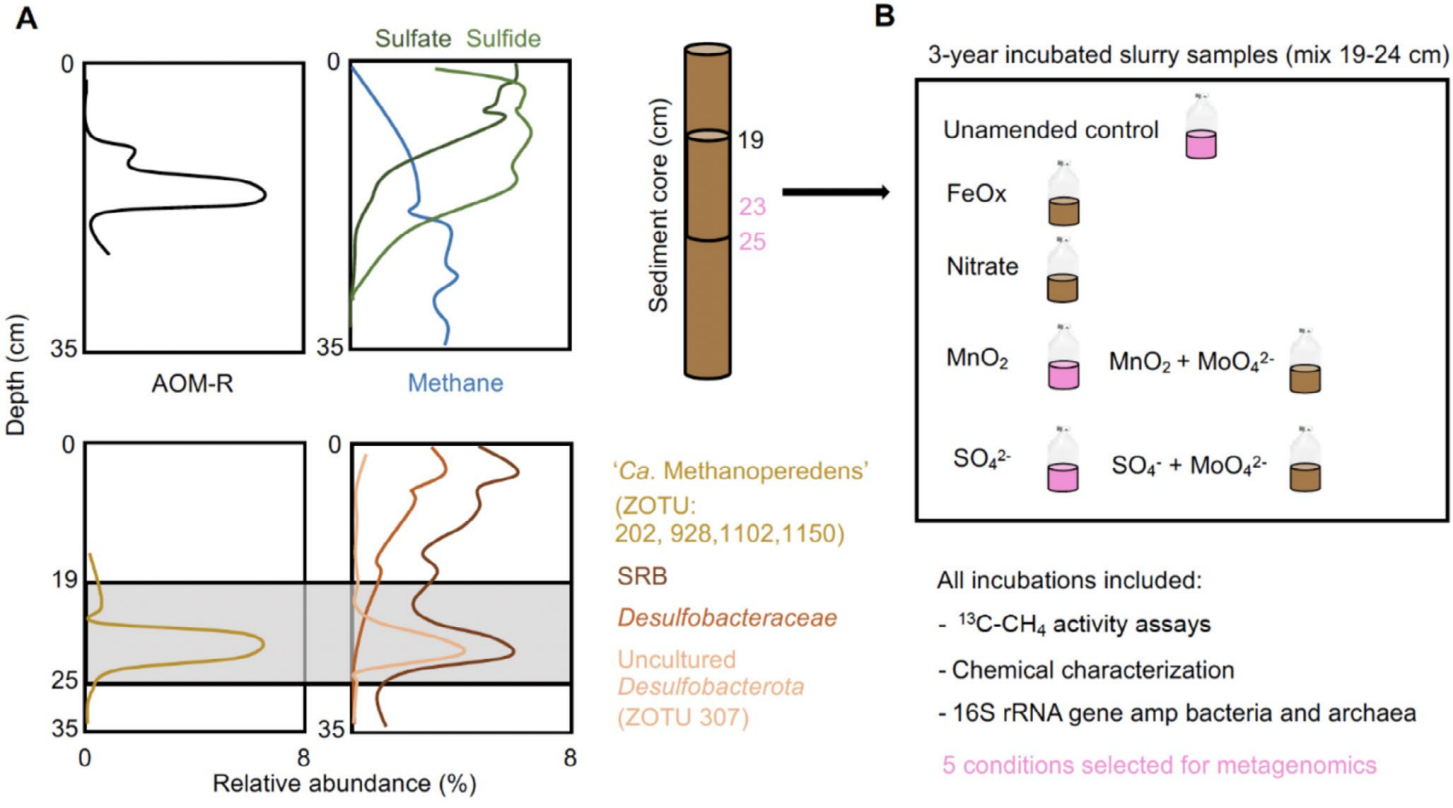
(A) Cartoon summarising previous anaerobic oxidation of methane (AOM) data from Lake Cadagno sediment (Su et al. 2020). Top: Depth‐specific in situ anaerobic oxidation of methane oxidation rates (AOM‐R) determined using ^13^C‐CH_4_ tracer whole‐core incubations, and profiles of porewater concentrations of dissolved methane, sulphate and sulphide. Bottom: Depth profiles of 16S rRNA gene amplicons (relative abundances) of ‘*Ca*. Methanoperedens spp.’ and SRBs in the sediment. (B) Overview of long‐term slurry incubation assays presented in the current study, including the conditions employed and the parameters analysed. The sediment samples and incubations selected for metagenomics are coloured in pink.

In Lake Cadagno sediments, rates of anaerobic oxidation of methane (AOM‐R), the abundances of ‘*Ca*. Methanoperedens’ and the uncultured *Desulfobacterota* ASVs peaked at intermediate depths (19–25 cm), where methane, sulphate, and sulphide were present at concentrations of 2–3 mM, ~100 μM and 300–600 μM, respectively (Figure 1A). Figure 1A provides a graphical representation of the relevant data aforementioned of figs. 1, 4, and suppl. fig. 4 from (Su et al. 2020). Based on these results we selected specific sediment layers for metagenomic sequencing (23 and 25 cm) and for further long‐term slurry incubations (depths 19–24 cm) (Figure 1B).

### 2.2 Long‐Term Slurry Incubations

We established static slurry incubations with fresh sediment material (depths 19–24 cm), collected from the centre of Lake Cadagno (46°33′01.89″ N, 8°42′42.04″ E) in October 2017. The sediment cores were stored upright with a few cm of overlying water in the cold room (4°C), and processed within 1 week. In an anoxic chamber with an N_2_ atmosphere, we mixed the sediment material with anoxic freshwater medium in a volume ratio of ~1:4, and dispensed 100 mL of this slurry into 250 mL serum bottles. We supplemented the slurries with either synthesised δ‐MnO_2_ (10 mM), ferrihydrite (Fe_5_O_8_H·H_2_O, 10 mM), or sulphate (4.8 mM) (Figure 1B). Finally, we purged the mixed slurries with He before injecting 20 mL ^13^C‐CH_4_ (99.8 atom %, Campro Scientific) to all incubations, including an unamended live control slurry. The incubations were closely followed for up to 4 months and presented previously (Su et al. 2020). Subsequently, they were fed twice more with ^13^C‐CH_4_ but were otherwise left unmonitored in the dark, within the anoxic chamber at 25°C, for an additional 32 months, with the goal of potentially enriching for slow‐growing anaerobic methanotrophs under different electron acceptor conditions.

### 2.3 Chemical Characterisation of Slurry Samples

At the end of the incubation time, we collected the liquid phase of the slurries and filtered it through 0.45‐μm filters for nutrient analysis. Sulphate was quantified by ion‐chromatography (940 Professional IC Vario, Metrohm, Switzerland), while Mn^2+^, Fe^2+^ and H_2_S were determined by ICP and photometry, as described previously (Cojean et al. 2020). DIC concentrations were quantified on a TOC analyser (Shimadzu, Corp., Kyoto, Japan). The carbon isotopic composition of DIC was determined by acidifying 1 mL filtered supernatant with 200 μL 85% H_3_PO_4_ in 12 mL He‐flushed exetainers. The δ^13^C of the released CO_2_ was determined in the headspace after overnight equilibration via a gas‐bench coupled to a Delta V Plus IRMS. The solid phase of incubation slurries was centrifuged and lyophilized prior to elemental analysis. Total carbon (TC) and total organic carbon (TOC, after acidification of the samples) as well as their δ^13^C values were determined by EA‐IRMS using a Delta V Plus IRMS and a ConFlow IV interface (Thermo Fisher Scientific, Bremen Germany). Stable carbon isotope values are reported in the conventional δ‐notation (in ‰) relative to the Vienna Pee Dee Belemnite standard (V‐PDB). Sulphate was quantified by ion‐chromatography (940 Professional IC Vario, Metrohm, Switzerland), while Mn^2+^, Fe^2+^ and H_2_S were determined by ICP and photometry, as described previously (Cojean et al. 2020).

### 2.4 DNA Extraction, Amplicon Sequencing and Metagenomics

We extracted DNA from the inocula sediments and the incubation slurries using the FastDNA Spin Kit for Soil (MP Biomedicals, city, country) and performed amplicon sequencing of 16S rRNA genes following the two‐step PCR approach (https://support.illumina.com/documents/documentation/chemistry_documentation/16s/16s‐metagenomic‐library‐prep‐guide‐15044223‐b.pdf) with primers 515F‐Y (5′‐GTGYCAGCMGCCGCGGTAA) and 926R (5′‐CCGYCAATTYMTTTRAGTTT‐3′) (Parada et al. 2016), targeting the V4 and V5 regions of the 16S rRNA gene. Amplicons were sequenced at the Genomics Facility Basel (University of Basel/ETHZ). Details of library preparation, sequencing, quality control and bioinformatical processing are described elsewhere (Su et al. 2023). We used SINTAX (v11.0.667_i86linux64, Edgar, 2016) and the SILVA 16S rRNA gene reference database v138.2 (Quast et al. 2013) to assign taxonomy to amplicon sequencing variants, and also to update the taxonomy of the previously described ‘*Desulfobulbaceae*’ SRB (ZOTU 307) in Su et al. (2020).

Furthermore, we selected two different sediment‐depths (23 and 25 cm) and three long‐term AOM incubations (unamended control, sulphate and manganese oxide) for downstream metagenomics (Figure 1B). Library preparation and sequencing of selected sediment and incubation DNA samples was performed at the Lausanne Genomic Technologies Facility, University of Lausanne, Switzerland (https://www.unil.ch/gtf). Paired‐end sequencing (150 cycles) was done on an Illumina HiSeq 2500 using the Nextera DNA Flex protocol, generating ~7 Gbp/sample for all samples except for the manganese oxides. Read quality was assessed with FASTQC v0.11.9 before and after quality trimming performed with BBDuk (BBTools v38.75). Trimmed reads were (co‐)assembled de novo using metaSPAdes v3.14.1 (Nurk et al. 2017) and mapped to assembled contigs using BBMap (BBTools v38.75) (Bushnell 2014). Five different assemblies were generated: (i) including sediment depths 23 and 25 cm reads, using the reads from the control (ii), sulphate (iii) and manganese oxides (iv) slurry incubations and (v) a bigger co‐assembly including all metagenome reads (i + ii + iii + iv) together. Contigs ≥ 1000‐bp length were used as template for read mapping. Sequence mapping files were handled and converted using Samtools v1.10, later used for binning with CONCOCT v2.1 (Alneberg et al. 2014), MaxBin2 v2.2.7 (Wu et al. 2016), and MetaBAT2 v2.12.1 (Kang et al. 2019).

Generated metagenome‐assembled genomes (MAGs) were dereplicated with DAS Tool v1.1.1 (Sieber et al. 2018) and taxonomically classified with the Genome Taxonomy Database Toolkit GTDB‐Tk v2.1.0 (Chaumeil et al. 2019). Metagenomic mapping statistics were generated via CheckM v1.1.2 (Parks et al. 2015). For metagenomic binning, taxonomical read‐recruitment assessment, and biogeography studies SingleM v0.16.0 and Sandpiper (https://sandpiper.qut.edu.au/) were used (May 2024), respectively (Woodcroft et al. 2024). MAG completeness and contamination was estimated with CheckM2 v1.0.1 (Chklovski et al. 2023). Open read frames (ORFs) in the MAGs were predicted and translated using Prodigal v2.6.3 (Hyatt et al. 2010). Proteins in MAGs were annotated with DRAM v1.5.0 (Shaffer et al. 2020) with default options, except min_contig_size at 1000 bp, and METABOLIC v4 (Zhou et al. 2022). Additionally, we searched for genes of interest in the annotation files via BLASTp, applying an e‐value cut‐off of 10^−5^. For a more accurate functional prediction of hydrogenases, we used the curated HydDB classifier http://services.birc.au.dk/hyddb/ (Søndergaard et al. 2016). To corroborate poorly annotated genes/proteins, manual curations were validated with the NCBI Batch Entrez Conserved Domains and InterPro (Blum et al. 2021). In this study, we consider microorganisms as ‘canonical SRBs’ if they encode the DsrD subunit of the dissimilatory sulphite reductase. This subunit has been identified as a marker for the enzyme's directionality, specifically for the reduction of sulphite to hydrogen sulphide (Diao et al. 2023). The search for proteins involved in SRB and ANME aggregate formation was done using DIAMOND with a list of protein InterPro IDs recovered from Murali et al. 2023 (IPR039662, IPR053783, IPR025295, IPR035903, PF01833) with e‐value and identity cut‐offs of 10^−5^ and 30%, respectively. Average amino acid or nucleotide identity (AAI or ANI) from MAGs was obtained using the FastAAI or FastANI‐matrix tool option (Rodriguez‐R and Konstantinidis 2016), using 90% AAI or 95% ANI for species cutoff (Jain et al. 2018; Konstantinidis et al. 2022).

### 2.5 Multi‐Heme *c*‐Type Cytochrome (MHC) Search and Domain Tree

We used the assembled metagenomes to investigate the presence and type of MHC on the five ‘*Ca*. Methanoperedens’ MAGs recovered together with the suspected syntrophic SRB ‘*Desulfobacterota* class QYQD01’. We first screened the selected MAGs for putative MHCs, identified by ORFs with ≥ 3 CXXCH motifs and used FeGenie v1.2 (Garber et al. 2020) annotations to differentiate Omcz nanowires, and BLASTp to identify Extracellular Cytochrome Nanowires (ECN). Subsequently, we used InterProScan v5.44‐79.0 (Jones et al. 2014) to identify domains classified as “multi‐heme cytochromes” from the identified ‘*Ca*. Methanoperedens’ MHC‐harbouring proteins. For this analysis, we also included reference bioreactor enrichment MAGs of ‘*Ca*. Methanoperedens’ with confirmed metatranscriptomic evidence for MHC expression under various conditions, including: *‘Ca*. Methanoperedens ferrireducens’ (Fe‐AOM) (Cai et al. 2018), *‘Ca*. Methanoperedens manganicus (Mn‐1)’ and *‘Ca*. Methanoperedens manganireducens (Mn‐2)’ (Mn‐AOM) (Leu et al. 2020), *‘Ca*. Methanoperedens nitroreducens’ Type Strain (electrode/nitrate/iron‐AOM) (Zhang et al. 2023) and *‘Ca*. Methanoperedens Vercelli’ (electrode‐AOM) (Ouboter et al. 2024). The identified MHCs domains were subtracted from the protein sequence and aligned using MAFFT v7.525 (Katoh and Standley 2013) via https://www.ebi.ac.uk/ with parameters –bl 62 –op 1.53 –ep 0.123 –reorder –retree 2 –treeout –maxiterate 2 –amino. Finally, we constructed a phylogenetic tree using IQ‐TREE v2.1.4 with function –s –st AA –m MFP –bb 1000 –nt AUTO, with a VT+R6 as best‐fit model based on Bayesian Information Criterion (BIC). All presented trees were annotated using iTOL v5 (Letunic and Bork 2021).

### 2.6 Extrachromosomal Elements (ECEs) Search

We additionally screened for putative ECEs in our five ‘*Ca*. Methanoperedens’ MAGs. Among the ECEs investigated, we included Borgs. For this, we first obtained a PFAM/InterPro database with dereplicated sequence representatives built from 40 different unique markers obtained from 17 different Borgs that appear to be associated with ‘*Ca*. Methanoperedens’ (Schoelmerich et al. 2024). Then we used DIAMOND v2.1.9.163 (Buchfink et al. 2015) to perform a BLASTp query search against our ‘*Ca*. Methanoperedens’ MAGs. For these annotations, we applied an e‐value cut‐off of 10^−5^ and a minimum amino acid identity of 30%.

We also searched for plasmids and viruses integrated into the ‘*Ca*. Methanoperedens’ MAGs described in this study by using VIBRANT (Kieft et al. 2020) and geNomad (Camargo et al. 2024) with default parameters. Initially, we identified 68 contigs as potential ECEs. To further investigate the nature of contigs, we classified them using specialised viral and phage annotation databases, including PHROGs (Terzian et al. 2021) pVOGs (Grazziotin et al. 2017), and VOGs (Bao et al. 2004). The e‐value and identity cut‐offs used for this annotation were 10^−5^ and 30%, respectively.

## 3 Results

### 3.1 Long‐Term Incubations With Different Electron Acceptors Preserve and Enrich ‘*Ca*. Methanoperedens’ as the Key Driver of AOM

The original carbon isotopic composition of the organic matter in the sediment used for the incubation experiment was −29.11‰ ± 1.99‰ **δ** ^13^C‐TOC. As we used ^13^C‐labelled methane to trace AOM in the slurry incubations with different electron acceptors (Table S1), any change towards more positive **δ** ^13^C‐TOC values is caused by the production of new biomass from ^13^C‐methane. Similarly, the production of enriched **δ** ^13^C‐DIC indicates active AOM. Accordingly, all incubations, including the unamended control slurry, showed AOM activity, as indicated by the different enrichment in ^13^C. Most strikingly, **δ** ^13^C‐DIC in the incubation with added MnO_2_ was significantly enriched in ^13^C relative to the control or bottles with sulphate addition. Mn‐AOM is a proton‐consuming, alkalinity‐producing metabolism (Table S1B) leading to low DIC concentrations in the MnO_2_ incubation due to MnCO_3_ precipitation, enhancing ^13^C label transfer into the solid phase. Both TOC and TC had much more positive δ^13^C values in the solid phase of the MnO_2_‐amended slurry compared to the incubation with sulphate addition or the unamended control. Notably, substantial amounts of sulphate were produced during the long‐term incubation with MnO_2_, while sulphide remained close to the detection limit. By comparison, high concentrations of sulphide (~0.5 mM) were detected in the slurry with sulphate addition at the end of incubation (Table S1). The addition of FeOOH enhanced organic matter degradation in the slurry but did not stimulate AOM more than in the unamended control. By 16S rRNA gene sequencing at the end of the incubation period, we find that all incubations were dominated by bacteria, predominantly composed of Chloroflexi and Proteobacteria, including the phylum Aminecenantes (Figure S1A,C). Within the archaeal fraction, Euryarchaeota dominated all incubations, particularly the manganese oxide incubation, followed by Woesearchaeota (Figure S1A,B). ‘*Ca*. Methanoperedens’ was retained under all conditions and was most enriched (19.2% relative abundance) in the manganese oxide incubation (Figure S1D). Conversely, canonical aerobic methane‐oxidising bacteria were detected only at very low relative abundances, below 0.004% (Figure S1E). All incubations selected for SRB closer to the *Syntrophaceae* rather than for the uncultured *Desulfobacterota* representative, previously described as *Desulfobulbaceae* (Figure S1F).

### 3.2 Co‐Occurrence of ‘Sed MAG Methanoperedens 1’ and SRB *Desulfobacterota* Class *QYQD01* Supports Possible Syntrophic Interactions

**FIGURE 2:**
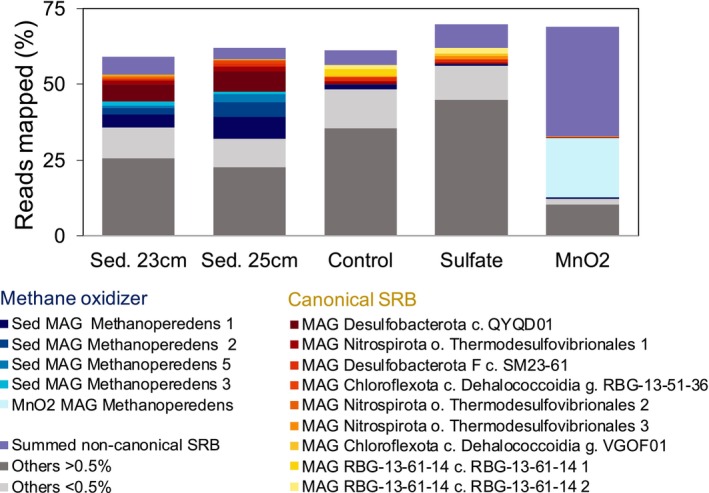
Percentage of mapped reads of top (above 0.5% for all metagenome sources) Metagenome Assembled Genomes (MAGs) analysed across the two sediment depths (23 and 25 cm) and the three incubations: Unamended control, sulphate, and manganese oxides. Subcategories include anaerobic methane oxidizers and canonical sulphate‐reducing bacteria (SRB) (containing *dsrABCD*) and non‐canonical SRB (containing *dsrABC*), as described in Diao et al. 2023. The ‘Others’ category is subdivided into all genomes that either exceeded 0.5% (dark grey) across all metagenome sources or fell below this threshold (light grey). The remaining reads constitute the unbinned fraction (not filling up to 100%).

To resolve the co‐occurrence and potential interaction of ANMEs and SRB in Lake Cadagno, a metagenome analysis was conducted, and five ‘*Ca*. Methanoperedens’ MAGs were retrieved (Figure 2). Four of the *‘Ca*. Methanoperedens’ MAGs were abundant in the original sediment while one MAG (labelled ‘MnO_2_ MAG Methanoperedens’, light blue) dominated the MnO_2_ incubation (Figure 2). The most abundant Methanoperedens MAG in the original sediment sample, ‘Sed MAG Methanoperedens 1’, contained a full‐length 16S rRNA gene that showed 100% identity to one of the sequence variants recovered by Su et al. (2020) (Figure 1, ZOTU202 and Figure S4 in original paper). ‘Sed MAG Methanoperedens 1’ sustained its dominance as the main anaerobic methane oxidizer for the unamended control and the sulphate incubation (Figure 2). The numbering of the ‘*Ca*. Methanoperedens’ MAGs is based on the metagenomic abundance of archaeal MAGs, from most to least abundant, in the co‐assembled sediment metagenome (23 cm depth) (Table S2). This resulted in ‘Sed MAG Methanoperedens 1, 2, 3 and 5’ (Figure 2), with ‘Sed MAG 4 c_Thermoplasmata’ being assigned number four.

The most prominent SRB MAG in the sediment belonged to the *Desulfobacterota* class *QYQD01*, with 5.6% to 6.7% of the reads in sediment 23 and 25 cm, respectively (Figure 2 and Table S3). ‘MAG Desulfobacterota class QYQD01’ showed 100% of 16S rRNA gene identity match with the most abundant uncultured Desulfobacterota ZOTU307, previously described as ‘uncultured *Desulfobulbaceae*’ (Su et al. 2020). The closest related microorganism to ‘MAG Desulfobacterota c. QYQD01’ in the NCBI database is the cultured *Desulfobacca acetoxidans* DSM11099, with an amino acid identity of 89.7% and 89% query cover. Consistent with the 16S rRNA gene analysis, the abundance of ‘MAG Desulfobacterota class QYQD01’ decreased to 0.2%, 0.05% and 0.03% in the unamended control, sulphate and manganese oxide incubations, respectively (Figures 2 and S1, Table S3). The unamended control and the sulphate treatment selected for canonical SRB from the order *Thermodesulfovibrionales*, as well as for the *Chloroflexota* phylum, genus *RBG‐13‐51‐26* or the phylum *RGB‐13‐61‐14* (Tables S3–S5).

### 3.3 MnO_2_ Amendment Selectively Enriched One ‘*Ca*. Methanoperedens’ sp. Representative From a Diverse Community

The most abundant MAG in the manganese oxide incubations, ‘MnO_2_ MAG Methanoperedens 1’ showed 99.7% 16S rRNA identity with one particular ‘*Ca*. Methanoperedens’ sp. ZOTU1150 (Su et al. 2020). When compared to MAGs ‘Sed MAG Methanoperedens 1,2,3 and 5’, the ‘MnO_2_ MAG Methanoperedens’ was rare in the original sediment, with around 0.007% relative abundance, and enriched to about 19.5% in the incubations amended with manganese oxide (Tables S3 and S6). These incubations also favoured the enrichment of non‐canonical SRB from the Chloroflexota phylum and the order *Anaerolineales*. These organisms lack the *dsrD* subunit but include sulphide oxidoreductases (*sqr*) (classified as non‐canonical SRB). The *Anaerolineales* contributed as much as 21% to the total metagenome (Table S6).

### 3.4 Recovered ‘*Ca.* Methanoperedens’ spp. Show EET Potential

**FIGURE 3:**
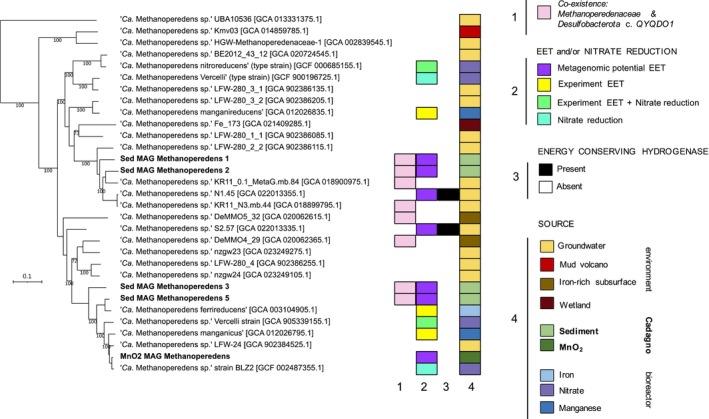
Phylogenomic tree of *Methanoperedeneacae* MAGs with Lake Cadagno representatives highlighted in bold. The tree has been generated using GTDB‐Tk classification tools with multiple sequence alignments of 53 archaeal phylogenetic markers. MAGs that were not in the GTDB database at the time of analysis were additionally included: GCA_018900975.1 and GCA_018899795.1 (Mehrshad et al. 2021), GCA_022013335.1 (Bell et al. 2022) and GCA_020062615.1 and GCA_020062365.1 (Casar et al. 2021). Extracellular electron transfer (EET) and presence/absence of hydrogenases are only indicated for MAGs with shown or suspected EET. Branch lengths represent the average number of amino acid substitutions per site. Bootstrap values are shown for > 70% branching support.

To uncover the main metabolic traits and biogeography of the five Lake Cadagno ‘*Ca*. Methanoperedens’ MAGs, we generated an annotated genome tree (Figure 3).

‘Sed MAG Methanoperedens 1 and 2’ were most closely related to a groundwater ‘*Ca*. Methanoperedens’ spp. (Figure 3). Conversely, ‘Sed MAG Methanoperedens 3 and 5’ and ‘MnO_2_ MAG Methanoperedens’ were more similar to ‘*Ca*. Methanoperedens’ spp. from bioreactor enrichments amended with nitrate or metal oxides. Among them, ‘MnO_2_ MAG Methanoperedens’ and the nitrate‐enriched ‘*Ca*. Methanoperedens BLZ2’ exhibit the greatest similarity, with an AAI of 92% (Figure 3 and Figure S2). Although phylogenetically closely affiliated with nitrate‐reducing species, none of the Lake Cadagno ‘*Ca*. Methanoperedens’ MAGs contained a nitrate reductase. We also screened for DNRA (dissimilatory nitrate reduction to ammonium) potential by searching for the functional genes for nitrite reductase (cytochrome c‐552) (*nrfA*) and cytochrome *c* nitrite reductase small subunit (*nrfH*). In the sediment‐associated *‘Ca*. Methanoperedens’ MAGs, we did not detect any *nrfAH* genes. However, in the ‘MnO_2_ MAG Methanoperedens’, we identified two *nrfA* genes (the catalytic subunit), but no *nrfH* was found, even in the unbinned fraction.

**FIGURE 4:**
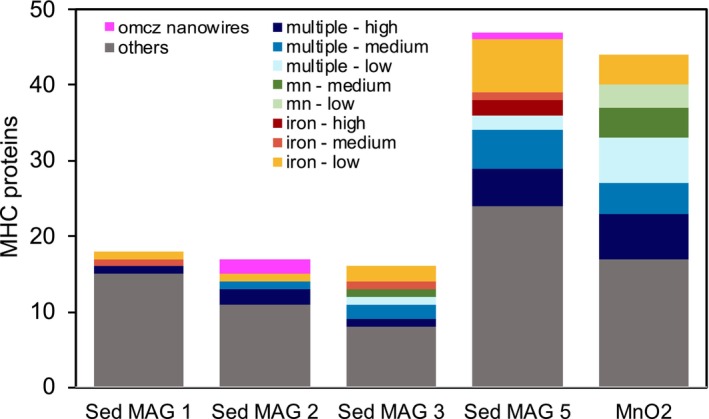
Multi‐heme *c*‐type cytochrome (MHC) protein counts in the Lake Cadagno ‘*Ca*. Methanoperedens sp.’ MAGs. Annotations are based on similarity (> 70% to domain MHC) to reference ‘*Ca*. Methanoperedens’ enrichment species that showed MHC expression under different electron accepting conditions—multiple (including electrode), manganese or iron oxides—and expression levels. ‘High’ expression refers to the top 10% of the MHC transcripts, ‘medium’ to the following 40%, and ‘low’ to the remaining MHC transcript expression. OmcZ‐like nanowire subunits are included as a separate category. The “Others” category includes MHC genes from this study that lack domain‐homology‐based expression links to reference ‘*Ca*. Methanoperedens’ MAGs. Comparing the similarity of MHC proteins among the Lake Cadagno ‘*Ca*. Methanoperedens’ spp. (Figure S4) revealed that they shared between 9 and 16 homologous MHC proteins. For details, see Figure S4.

All Lake Cadagno ‘*Ca*. Methanoperedens’ MAGs included the full reverse methanogenesis pathway for anaerobic oxidation of methane and lacked cytosolic or energy conserving hydrogenases (Ech) (Table S7). Closer inspection of the Lake Cadagno ‘*Ca*. Methanoperedens’ MAGs revealed the potential for EET via MHC proteins or OmcZ‐like subunits (Figures 3 and 4).

### 3.5 Distinct Multi‐Heme *c*‐Type Cytochrome (MHC) Assigned to Lake Cadagno ‘*Ca.* Methanoperedens’ spp.

Intrigued by the high AOM activity in the manganese oxide incubation and the enrichment of ‘MnO_2_ MAG Methanoperedens’, we analysed the metagenomes carefully for putative EET mechanisms. We investigated the type and similarity of MHC proteins plus their OmcZ‐like subunits putatively forming conductive nanowire proteins (Figures 4, S3 and S4, Table S8). OmcZ proteins are derived from the well‐characterised electroactive model species *Geobacter sulfurreducens* . *G. sulfurreducens* produces extracellular *c*‐type cytochromes with stacked heme arrangements that polymerise into filaments, enabling long‐range electron transport. Three types of conductive filaments have been described for *G. sulfurreducens* : OmcS (hexaheme) and OmcE (tetraheme), which form linear heme‐stacked filaments and, OmcZ (octaheme), which forms filaments with branching, stacked hemes (Wang et al. 2022). These structures are commonly referred to as microbial nanowires because of their role in facilitating extracellular electron transfer to insoluble electron acceptors or electrodes in bioelectrochemical systems (Yalcin et al. 2020). The ‘MnO_2_ MAG Methanoperedens’ and ‘Sed MAG Methanoperedens 5’ showed almost twice the amount of MHC proteins compared to ‘Sed MAG Methanoperedens 1, 2 and 3’. ‘MnO_2_ MAG Methanoperedens’ and ‘Sed MAG Methanoperedens 5’ had 44 and 47 MHCs, respectively (Figure 4). Only ‘Sed MAG Methanoperedens 2 and 5’ encoded two and one OmcZ‐like subunits possibly forming nanowire proteins, respectively. The other ‘MnO_2_ MAG Methanoperedens’ did not contain *omcZ* like genes. Instead, ‘MnO_2_ MAG Methanoperedens’ (contig_13_120) contained an Extracellular Cytochrome Nanowire (ECN) (Baquero et al. 2023) that shared 98% BLASTp identity with ‘*Ca*. Methanoperedens BLZ2’ ECN [WP_097300794.1], matching the high AAI (92%) of both MAGs (Figure S2).

In Ouboter et al. (2024) and Leu et al. (2020), several gene clusters are described to be involved in EET by ‘*Ca*. Methanoperedens’. Therefore, we assessed which MHC genes of the Lake Cadagno ‘*Ca*. Methanoperedens’ MAGs resembled those clusters. We find that more than half of the MHC proteins in ‘MnO_2_ MAG Methanoperedens’ and in ‘Sed MAG Methanoperedens 3 and 5’ showed high homology with MHC proteins expressed in the reference ‘*Ca*. Methanoperedens’ spp. (Figure 4). For example, in ‘MnO_2_ MAG Methanoperedens’, 7 out of the 44 MHC proteins were related to those highly expressed in ‘*Ca*. Methanoperedens’ spp. grown with manganese oxides as the electron acceptor, while 4 were related to MHC proteins with low expression under iron‐oxide‐reducing conditions. In ‘Sed MAG Methanoperedens 5’, 9 out of the 49 MHC proteins corresponded to MHC proteins expressed in the iron‐AOM ‘*Ca*. Methanoperedens’ enrichment, with the majority showing low expression levels, and a few matching those with medium or high expression (Figure 4). Furthermore, ‘Sed MAG Methanoperedens 5’ *omcZ*‐like genes appeared to be clustering close to the highly expressed *omcZ*‐like gene in ‘*Ca*. Methanoperedens ferrireducens’ performing Fe‐AOM (Figures 4 and S3).

We also examined the presence of additional EET electroconductive structures. We identified type IV pilus assembly proteins in all Lake Cadagno ‘*Ca*. Methanoperedens’ MAGs. Furthermore, we investigated the formation mechanisms of extracellular polymeric substances (EPS), as these could facilitate the development of ANME/SRB consortia through the formation of cell aggregates and biofilms, as described by Murali et al. (2023). This search revealed the presence of cohesion subunit Scc3/SA, dockerin‐like domain proteins and extracellular Contractile Injection Systems (eCIS) across all ‘*Ca*. Methanoperedens’ MAGs investigated, which either could help in the cellular adhesion or intercellular communication between organisms (Table S9).

### 3.6 Presence of Mobile Genetic Elements (MGEs) in Lake Cadagno's ‘*Ca*. Methanoperedens’

To assess the metabolic flexibility and putative horizontal gene transfer (HGT) in the Lake Cadagno ‘*Ca*. Methanoperedens’ MAGs, we investigated the presence of ECEs. In this context, we looked at the presence of Borgs, which have been described as novel giant ECEs, that cannot be classified as virus or plasmid. They have been shown to be associated with ‘*Ca*. Methanoperedens’ and are believed to augment the metabolic potential of ‘*Ca*. Methanoperedens’ as they have a propensity to assimilate genes, for example, encoding for key metabolic pathways such as anaerobic methane oxidation, extracellular electron transfer, or stress resistance (Al‐Shayeb et al. 2022; Schoelmerich et al. 2024). More recently, 40 unique putative Borg markers have been described (Schoelmerich et al. 2024), which we employed against our ‘*Ca*. Methanoperedens’ MAGs. Our search resulted in 16 out of 30 family domain putative Borg marker proteins (Tables S13 and S14), suggesting the absence of previously described Borg ECEs in our samples.

We screened for additional mobile genetic elements (MGEs) in the ‘*Ca*. Methanoperedens’ MAGs (Tables S15–S17), and identified 64 contigs as potential MGEs (Table S18). To assess whether any of these were viral, we screened for specific structural proteins (e.g., capsids) but found no positive hits (Tables S19–S21). We then looked for alternative signature proteins, including integrases and transposases, detecting seven and six hits, respectively (Tables S18–S21). Finally, in those integrases and transposases harbouring contigs, we checked for the presence of terminal repeats, an indicator of MGE completeness, and identified two instances in ‘Sed MAG Methanoperedens 1’ (contig_3571) and ‘Sed MAG Methanoperedens 2’ (contig_21328) (Table S18).

We also assessed the possible role of MGE in HGT of key genes implicated the adaptation of SRB to a syntrophic partnership with ANME, as proposed by Murali et al. (2023). However, proteins encoding structures responsible for aggregate formation between ANME and SRB (Table S9) were not identified in any of the 64 potential MGEs investigated (Table S18). This suggests that MGEs do not influence the establishment of this partnership.

### 3.7 Phylogeny, Biogeography and Genomic Analysis of the Putative S‐AOM Partner *Desulfobacterota* Class *QYQD01*

**FIGURE 5:**
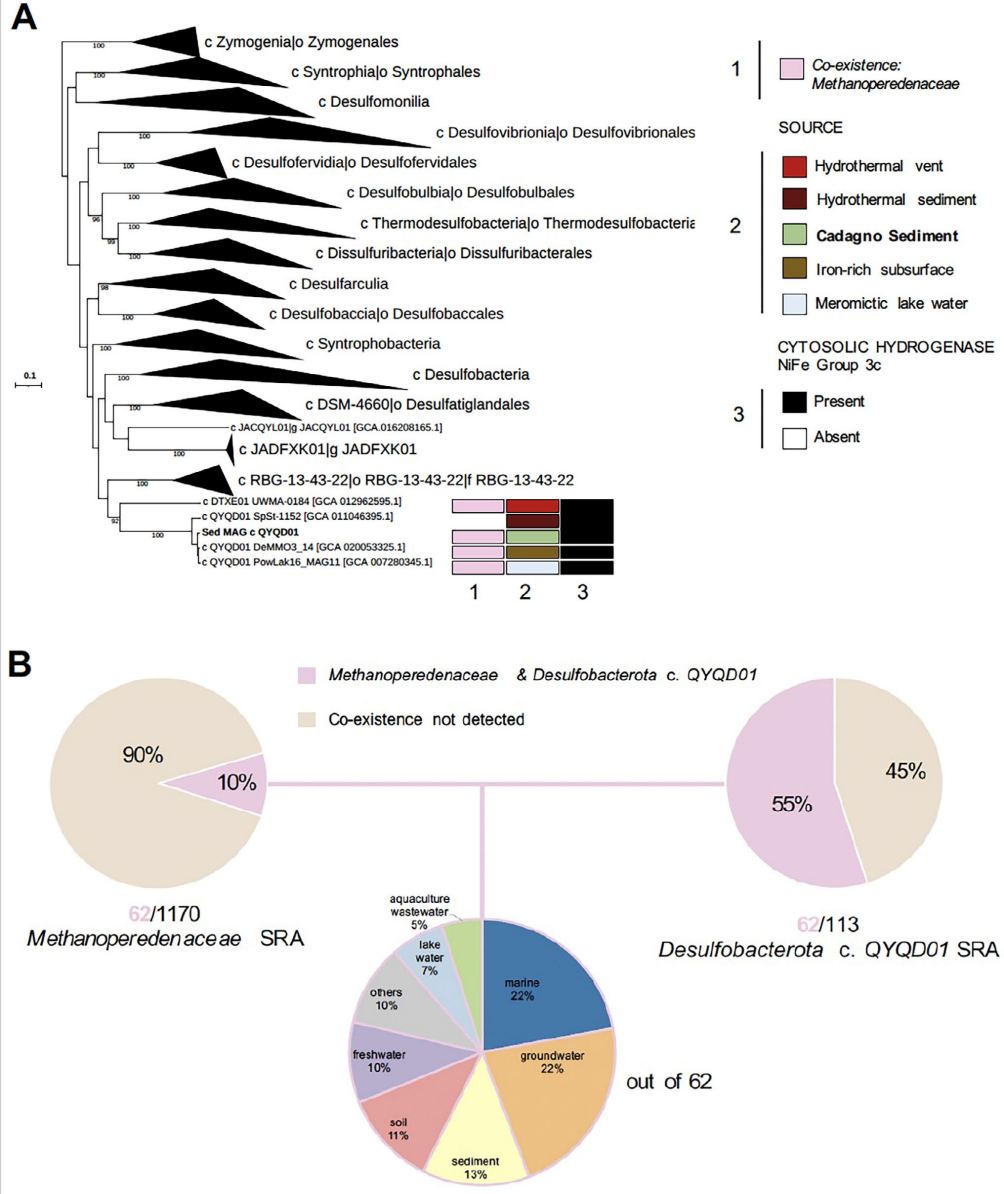
(A) Phylogenomic tree of the *Desulfobacterota* phylum based on Metagenome Assembled Genomes (MAG) with collapsed clades except for class QYQD01 and classDTXE01. The tree was generated via GTDB‐Tk classification tools using multiple sequence alignments via concatenation of 120 bacterial phylogenetic markers. One MAG was included in addition to the genomes retrieved from the GTDB database: GCA_020053325.1 (Casar et al. 2021). Bootstrap values are shown for > 70% branching support. Branch length represents the average number of amino acid substitutions per site. (B) From top left to right, sequence read archive (SRA) hits for *Methanoperedeneaceae* family and *Desulfobacterota* class QYQD01. Colours indicate percentages of Sequencing Read Archive (SRA) entries where both groups were found in the same data set (pink) or not (brown). Bottom pie‐chart displays SRAsource from where both groups were found present.

After the striking co‐occurrence of the SRB *Desulfobacterota* class *QYQD01* with ‘*Ca*. Methanoperedens’ in Lake Cadagno sediments, we took a closer look at the *Desulfobacterota* class *QYQD01* MAG. We observed that the class *QYQD01* and the neighbouring class *DTXEO1* form a distinct clade within the *Desulfobacterota* phylum, with the order *Desulfatiglandale*s representing the most closely related cultivated SRB (Figure 5A). We identified three additional *Desulfobacterota* class *QYQD01* genomes in the GTBD, two of which, DeMMO_14 (Deep Mine Microbial Observatory) and PowLak16 (Powell Lake), classified as the same species (Figure S5), and all three co‐occurred with *Methanoperedenaceae* (Figures 5A and S6). Both the *Desulfobacterota* class *QYQD01* and *DTXEO1* genomes were recovered from diverse environments such as hydrothermal vents, sediments, meromictic lakes or iron‐rich subsurface waters. Notably, in the iron‐rich subsurface water metagenome, Desulfobacterota MAG (DeMMO3_14) was found alongside a ‘*Ca*. Methanoperedens’ MAGs (DeMMO4_29 and DeMMO5_32; Figure 3).

Using the tool Sandpiper, we screened the sequence read archive (SRA) for metagenomic reads of *Desulfobacterota* class *QYQD01*, to assess whether this class has been observed alongside *Methanoperedenaceae* before (Tables S10 and S11). Our search resulted in 62 metagenomes where their co‐existence was detected (Figure 5B). A quarter of the sequence SRA descriptions belonged to marine and groundwater systems, followed by sediment, soil, freshwater, as well as lake water, and aquaculture waste (Figure 5B). We determined the metagenomic coverage of reads from *Methanoperedenaceae* and *Desulfobacterota* class *QYQD01* in the 62 metagenomes in the ecosystems where they co‐occurred (Figures 5B and S6A). The metagenomic coverage of *Methanoperedenaceae* to *Desulfobacterota* class *QYQD01* was much higher in groundwater systems (0 to 360) than in marine ecosystems (0 to 25) (Figure S6A). We also found that *Methanoperedenaceae* co‐existed with Desulfobacterota (class *QYQD01*) in about 70% of the groundwater samples from Finland's Olkiluoto Island, and in 21% of those from an arsenic‐contaminated site in China (Figure S6B). The Olkiluoto Island deep subsurface sample source was also shared by the *Methanoperedenaceae* MAGs presented in the above genome tree, labelled as KR11_0.1_MetaG.mb.84 and KR_11_N3.mb.44 (Mehrshad et al. 2021) and N1.45 and s2.57 (Bell et al. 2022) (Figure 3). The marine metagenome samples were dominated by mangrove, seagrass, estuary or oilfield sediments (50%), followed by the Deep Horizon Spill Sediment (21%) (Figure S6B).

We additionally investigated the genetic features that could be indicative of a potential interdependency of the *Desulfobacterota* class *QYQD01* and ‘*Ca*. Methanoperedens’ spp. to sustain syntropy. The recovered *Desulfobacterota class QYQDO1* MAG contained the full respiratory sulphate reduction pathway that all ‘*Ca*. Methanoperedens’ spp. lacked. We did not find evidence for sulphur disproportionation potential in the Desulfobacterota *class QYQDO1* MAG. Conversely, we found a total of 18 MHC encoded in the ‘MAG Desulfobacterota class QYDO1’ (Figure 5 and Table S8). The largest MHC of ‘MAG Desulfobacterota class QYDO1’ was encoded within an operon comprising four adjacent sequences. These sequences encoded two MHC proteins—one with 12 heme binding motifs and another with 26 heme binding motifs—including a peptidyl‐prolyl isomerase (or PPIase) sequence containing 5 heme binding motifs, and a small ORF of unknown function. The second gene cluster encoding large MHCs encoded two cytochromes with 11 and 12 heme‐binding motifs, respectively, and an upstream Adenosine monophosphate (AMP) nucleoside‐encoding protein plus two other cytochrome C assembly and biogenesis proteins (Table S8).

Additionally, we also considered the syntrophic lifestyle of SRB associated with marine ANME, and included putative extracellular polysaccharides and protein complexes that could aid in the interaction with ‘*Ca*. Methanoperedens’ as described in Murali et al. (2023) (Table S12). Consistent with our observations on the aggregate formation mechanisms of ‘*Ca*. Methanoperedens’ (Table S9), the presence of certain marker genes in the ‘MAG Desulfobacterota class QYQD01’ supports ANME/SRB DIET potential and communication (Table S12). These structures include type IV pili (EET mechanism), the type VI secretion system (for intercellular communication), and potential adhesins for cellular adhesion, such as the trimeric autotransporter adhesin YadA‐like head domain. Several proteins with doubled CXXCH motifs were also detected (Table S12).

We also analysed some hallmark genomic traits that could suggest a free‐living lifestyle of *Desulfobacterota* class QYQD01 as a putative autotrophic SRB. The ‘MAG Desulfobacterota class QYQD01’ included the potential for carbon fixation via the Wood–Ljungdahl pathway. We also recovered non‐energy conserving cytosolic hydrogenases (NiFe group 3c) for our study's MAG and the three other *Desulfobacterota* class *QYQD01* MAGs included in the genome tree (Figure 5A). For our ‘MAG Desulfobacterota class QYQD01’, the accessory hydrogenase subunits (*hyd*) appeared together in a separate region of the genome forming a different operon that was not adjacent to the large and small NiFe group 3c subunits.

Other observations included the lack of phosphate acetyltransferase and acetate kinase for the conversion of acetyl‐CoA to acetate and nitrate or nitrite reductase (Table S7).

## 4 Discussion

This study deepens our knowledge of the poorly explored potential syntrophic interaction between ‘*Ca*. Methanoperedens’ spp. and the *Desulfobacterota* class *QYQD01* in Lake Cadagno sediments (Figures 1, 2, and S1). After screening of the SRA, we also discovered a potentially widespread co‐existence of *Desulfobacterota* class *QYQD01* and ‘*Ca*. Methanoperedens’ spp. (Figures 3 and 5) in many groundwater and marine ecosystems (Figures 5B and S6).

Long‐term slurry incubations under different electron acceptor conditions resulted in the enrichment of ‘MnO_2_ MAG Methanoperedens’ constituting up to about 19.5% (Figure 3) of the microbial community, which aligns well with the ~20% relative abundance of one of the ‘*Ca*. Methanoperedens’ ASVs (ZOTU1150). All incubations showed an apparent reduction of putatively syntrophic ‘MAG *Desulfobacterota* class *QYQD01’* (Figure 2). This is especially surprising for the sulphate amendment as we did observe a stimulation of SRB in the identical short‐term incubations (Su et al. 2020). We suspect that the accumulation of sulphide (489 μM, Table S1) slowed growth of ‘*Ca*. Methanoperedens’, similar to the sulphide toxicity‐related inhibition observed in other ANMEs (Dalcin Martins et al. 2022). In the Lake Cadagno sediment core investigated by Su et al. (2020), maximum abundance of ‘Ca. Methanoperedens’ was observed in zones where sulphide concentrations were < 500 μM. In natural environments, sulphide toxicity may be mitigated if porewater sulphide remains low—either through chemical reaction with Fe/Mn minerals in the sediment, by biological activity, or physical processes such as advective porewater transport.

Recently, Group III Dsr‐LP sulphite reductases have been linked to sulphide toxicity in a ‘*Ca*. Methanoperedens BLZ2’ enriched culture (Echeveste Medrano et al. 2025) and to sulphite detoxification in the model methanogen *Methanococcus maripaludis* (Day et al. 2024). In the current study, we recovered one Group III Dsr‐LP protein in ‘Sed MAG Methanoperedens 1’ and ‘Sed MAG Methanoperedens 5’, and two in ‘Sed MAG Methanoperedens 2’ and the enriched ‘MnO_2_ MAG Methanoperedens’ representative. Group III Dsr‐LP have been only described in some methanogens and ‘*Ca*. Methanoperedens’ spp., but not in marine ANMEs (Yu et al. 2018). In line with Echeveste Medrano et al. 2025, we hypothesize that the role of Group III Dsr‐LP sulphite reductases in the recovered MAGs is most likely linked to sulphite detoxification, given the absence of both nitrate and nitrite reductases (*nrfAH*) in the sediment ‘*Ca*. Methanoperedens’ MAGs, and the high sulphate‐to‐nitrate availability.

Our data indicate that ‘Sed MAG Methanoperedens 1’ and ‘Sed MAG Methanoperedens 2’ are the most plausible candidates to engage in a syntrophic interaction with *Desulfobacterota* class *QYQD01*. They clustered more closely with environmental groundwater ‘*Ca*. Methanoperedens’ spp., that have been found in sulphate‐rich lake and subsurface water, and to some that have also been found to co‐occur with the *Desulfobacterota* class *QYQD01* (Figure 3). Conversely, ‘MnO_2_ MAG Methanoperedens’, ‘Sed MAG Methanoperedens 3’ and ‘Sed MAG Methanoperedens 5’, are more closely related to metal‐reducing ‘*Ca*. Methanoperedens’ species (Figure 3). The putative MHC‐enabled EET mechanisms of ‘MnO_2_ MAG Methanoperedens’ and ‘MAG Methanoperedens 5’ resemble most those of ‘*Ca*. Methanoperedens’ spp. enriched in cultures amended with metal (manganese and iron) oxides or other electron acceptors (electrode and nitrate) enrichments (Figure 3) (Cai et al. 2018; Leu et al. 2020; Zhang et al. 2023; Ouboter et al. 2024). In contrast, the predicted syntrophic ‘Sed MAG Methanoperedens 1’ and ‘Sed MAG Methanoperedens 2’ harbour less homologous MHC proteins to that of known species, potentially indicating novel functionality (Figure 4 and Figure S3). These two candidate MAGs were also more closely related to the ones observed in the Olkiluoto Island deep subsurface (Figure 3).

Our co‐occurrence *Desulfobacterota* class *QYQD01* and *Methanoperedenaceae* biogeography study revealed a high correlation of *Methanoperedenaceae* to *Desulfobacterota* class *QYQD01* in groundwater systems (Figures 5B and S6). This putative syntropy could represent a survival strategy for *Methanoperedenaceae* to dispose of electrons from methane oxidation when the environmental metal oxide pools get depleted. One of the iron‐rich subsurface metagenomic studies included genomes of both *Desulfobacterota* class *QYQD01* and two ‘*Ca*. Methanoperedens’ spp., labelled as DeMMO (Figures 4A and 5A) (Casar et al. 2021). They observed several genes encoding for proteins involved in iron cycling in six different fracture fluids with varying chemistry. Here, one clear difference between sites was the high sulphate concentration, ranging from 0.88 to 42.79 mM. The *Methanoperedenaceae* appeared to contribute to iron reduction the most in site D6, where sulphate was the highest among all sites. In Casar et al. (2021), SRB genomes from the ‘*Desulfobacterales*’ taxa were also suggested to contribute with 2%–4% to the relative metagenomic iron reduction. The second *Desulfobacterota* class *QYQD01* genome (PowLake16_MAG 11) (Figure 5A) was retrieved from the meromictic Lake Powell, and co‐occurred also with ‘*Ca*. Methanoperedens’ (Figure S6A, indicated with an arrow) (Haas et al. 2019).

We further explored ‘MAG Desulfobacterota class QYQD01’ for genomic features that could be indicative of a syntrophic lifestyle with ‘*Ca*. Methanoperedens’ spp. Our observations resulted in high congruence with genomic traits described for syntrophic SRB partners of marine ANME (Skennerton et al. 2017; Murali et al. 2023), including: the putative loss of Ech hydrogenase, type IV pili formation and type VI secretion system, presence of adhesins and the conservation of large MHCs for DIET. In this regard, the largest MHC in our ‘MAG Desulfobacterota class QYQD01’ was contained in a four‐sequence operon structure that resembled the one described for SEEP‐SRB 1 marine SRB as well as homologous organisms presented in the same study (Sed_Bac_MAG_1_p_Desulfobacterota_c_QYQD01_contig_10778 ORF, 1 to 4) (Tables S7 and S8). The analysed SEEP‐SRB MHC operon structure included one 26 and another 16 heme *c*‐type cytochromes followed upstream by a PPIase domain and downstream by a beta propeller fold protein (Skennerton et al. 2017). Our ‘MAG Desulfobacterota class QYQD01’ also included a 26 and 12 (not 16) heme *c*‐type cytochrome with a PPIase domain but since the contig in our study/MAG was broken downstream, we were not able to conclude whether this operon also included a gene encoding a beta‐propeller fold protein. Compared to the genomic signature traits of marine SRB associated with ANME, our SRB lacked Ech hydrogenases. This observation contrasts with the NiFe group 1b hydrogenase reported for the Olkiluoto Island Deep subsurface in Finland, where S‐AOM was proposed via *Desulfobacterales* family *ETH‐SRB1* and ‘*Ca*. Methanoperedens’ spp. N1.45 and S2.57 (Figure 3). For the here described putative syntrophic SRBs, we recovered cytosolic NiFe group 3c hydrogenases.

Murali et al. 2023 assembled widespread syntrophic marine SRB clades that partner with ANME to perform S‐AOM and identified traits suggestive of adaptation to a syntrophic lifestyle, such as the ability for biofilm formation, intercellular communication, or for some Seep‐SRB1a, a nutritional dependency on ANME based on the lack of a cobalamin synthesis pathway. Our study's *Desulfobacterota* class *QYQD01* clustered closely to marine syntrophic group Seep‐SRB1g in the presented *Desulfobacterota* genome tree (Skennerton et al. 2017, Murali et al. 2023). For this comparative genomics study, *Desulfobacterales* family ETH‐SRB1 (refered to as Seep‐SRB1a sp.1) was also considered as a syntrophic SRB, aligning with the presented S‐AOM in co‐abundance with ‘*Ca*. Methanoperedens' spp. N1.45 and S2.57 in Olkiluoto Island deep subsurface in Finland (Bell et al. 2022; Murali et al. 2023).

Our investigation into ECEs in ‘*Ca*. Methanoperedens’ produced inconclusive results regarding the presence of Borgs. Specifically, the markers used to identify Borgs (Schoelmerich et al. 2024) did not provide clear evidence of their presence in the recovered genomes. Only 17 out of the 30 Borg family protein markers were detected (Tables S13 and S14) so the presence of previously identified Borgs seems unlikely. Whether those 17 Borg family marker proteins belong to as of yet unidentified Borgs needs to be further investigated. Additionally, only two non‐viral MGE belonging to ‘Sed MAG Methanoperedens 1’ and ‘Sed MAG Methanoperedens 2’ were conclusively identified (Table S18).

To conclude, we report a widespread co‐occurrence of ‘*Ca*. Methanoperedens’ spp. and *Desulfobacterota* class *QYQD01*, likely performing S‐AOM in sulphate‐rich freshwater lakes, but also appear to be present in groundwater and even marine systems. We suggest putative MHC‐proteins of ‘*Ca*. Methanoperedens’ that could engage via EET in a syntrophic interaction and present metabolic adaptations and phylogenomic placement of *Desulfobacterota* indicative of a syntrophic lifestyle with ANME archaea. Future efforts should focus on S‐AOM‐targeted enrichments of ‘*Ca*. Methanoperedens’ and syntrophic SRB in bioreactors inoculated with sediment from Lake Cadagno. Metatranscriptomic and metaproteomic studies could be conducted on the sediment to determine which ‘*Ca*. Methanoperedens’ and SRB species are most active, identify the preferred MHC proteins, and investigate whether Group III Dsr‐LP plays a role in sulphide‐derived sulphite detoxification.

## Author Contributions

Conceptualization: M.J.E.M. (equal), G.S. (equal), J.Z. (equal). Data curation: M.J.E.M. (equal), G.S. (equal), P.L. (supporting). Formal analysis: M.J.E.M. (equal), G.S. (equal), L.A.B. (supporting). Funding acquisition: J.Z. (equal), C.U.W. (equal), I.S.A. (equal), M.S.M.J. (equal), G.S. (supporting). Investigation: M.J.E.M. (equal), G.S. (equal), J.Z. (equal). Methodology: M.J.E.M. (equal), G.S. (equal), J.Z. (equal), P.L. (supporting). Resources: M.J.E.M. (equal), G.S. (equal), J.Z. (equal), P.L. (supporting). Supervision: J.Z. (equal), C.U.W. (equal), I.S.A. (equal), M.S.M.J. (equal). Visualization: M.J.E.M. (lead), G.S. (supporting). Writing – original draft preparation: M.J.E.M. (lead), J.Z. (supporting). Writing – review and editing: M.J.E.M. (equal), G.S. (equal), J.Z. (equal), C.U.W. (equal), P.L. (supporting), L.A.B. (supporting), I.S.A. (supporting), M.S.M.J. (supporting).

## Conflicts of Interest

The authors declare no conflicts of interest.

## Supporting information


**Data S1.** Supporting Figures.

**Table S2.** Completeness, contamination, bin size and metagenomic based % of mapped reads from the MAGs from sediment depths 23 and 25 cm co‐assembly.
**Table S3.** Metagenomic based % of mapped reads of 5 co‐assembled metagenomes from sediment depths 23 and 25 cm plus incubations from belonging control, MnO_2_ (manganase incubation) and sulphate.
**Table S4.** Completeness, contamination, bin size and metagenomic based % of mapped reads from the MAGs from the control incubation assembly.
**Table S5.** Completeness, contamination, bin size and metagenomic based % of mapped reads from the MAGs from the sulphate incubation assembly.
**Table S6.** Completeness, contamination, bin size and metagenomic based % of mapped reads from the MAGs from the MnO_2_ incubation assembly.
**Table S7.** DRAM based metagenome assembled genomes (MAGs) annotations from the metagenome co‐assembly from sediment depths 23 and 25 cm and live control, sulphate and manganse oxide incubations.
**Table S8.** Inteproscan results and Fegenie iron reduction protein categorization of studied ‘*Ca*. Methanoperedens’ and Desulfobacterota QYQD01 MHCs, including MHC counts.
**Table S9.** Results on Murali et al. 2023 (original fig. 6) hits on ANME/SRB formation protein aggregrates. ‘*Ca*. Methanoperedens’ MAG hits.
**Table S10.** SingleM‐based sandpiper results on *Methanoperedeneaceae* family.
**Table S11.** SingleM‐based sandpiper results on Desulfobacterota class QYQD01.
**Table S12.** Results on Murali et al. (2023) (original fig. 6) hits on ANME/SRB formation protein aggregrates. ‘Desulfobacterota QYQD01’ MAG hits.
**Table S13.** DIAMOND BLAST hits using PFAM/INTERPRO ids generated from BORG markers described in Schoelmerich et al. 2024. E‐values are equal or below 10–5 and with a minimum alignment of 30%.
**Table S14.** Presence/absence of BORGs marker proteins (against respective PFAM/INTERPRO ids) in ‘Lake Cadagno’ ‘*Ca*. Methanoperedens’ MAGs. E‐values are equal or below 10–5 and with a minimum alignment of 30%.
**Table S15.** Mobile genetic element (MGE) screening on ‘*Ca*. Methanoperedens’ MAGs: VIBRANT output.
**Table S16.** Mobile genetic element (MGE) screening on ‘*Ca*. Methanoperedens’ MAGs: geNOMAD output (plasmid).
**Table S17.** Mobile genetic element (MGE) screening on ‘*Ca*. Methanoperedens’ MAGs: geNOMAD output (virus).
**Table S18.** Putative 64 Mobile Genetic Elements (MGEs) identified in ‘*Ca*. Methanoperedens’ genomes: analysis based on presence/absence of transposases, integrases and terminal repeats.
**Table S19.** Prokaryotic Virus Remote Homologous groups search via PHROGs.
**Table S20.** Prokaryotic Virus Orthologous Groups search via pVOGs.
**Table S21.** Viral protein clusters groups search via VOGs.

## Data Availability

Raw sequence data for 16S rRNA amplicon sequencing are made available at NCBI under the BioProject ID PRJNA497531 with the accession numbers SRR15689035‐SRR15689041. Raw metagenomic data and MAGs recovered from the sediment and manganese oxide incubation co‐assemblies have been uploaded to the European Nucleotide Archive with project number PRJEB81702. Tables S2–S21 (as a single spreadsheet) have been deposited at Zenodo under the following DOI: https://doi.org/10.5281/zenodo.14055788.
