# Supplementary material for: Methanotrophic Flexibility of ‘Ca. Methanoperedens’ and Its Interactions With Sulphate‐Reducing Bacteria in the Sediment of Meromictic Lake Cadagno
Source: Environ Microbiol. 2025 Jul 10;27(7):e70133. doi: 10.1111/1462-2920.70133 (PMC12246660; doi:10.1111/1462-2920.70133)
Supplement: Supplementary file 1 — Data S1. Supporting Figures. [file EMI-27-e70133-s001.docx]

**Appendix**

**Supplementary Table 1 A.** Chemical characterization of aqueous and solid phases at the end of the long-term slurry incubations. Homogenized Lake Cadagno sediment from the 19-24 cm depth layer was used as the inoculum for the anaerobic slurry incubations and amended with ^13^C- methane and different electron acceptors. The unamended control is a live control, prepared as the other incubations but without additional electron acceptor added. **B** Stoichiometries of anaerobic methane oxidation with different electron acceptors used in incubations.

| **A** | | | |  | | | | Unamended  Control | | | | MnO_2_  10 mM | | | FeOx  10 mM | | | Sulfate  4.8 mM | | |  |
| --- | --- | --- | --- | --- | --- | --- | --- | --- | --- | --- | --- | --- | --- | --- | --- | --- | --- | --- | --- | --- | --- |
| Aqueous phase | | | | | Sulfate (µM) | | | | 0.6 | | | 272 | | | | 0.2 | | | 105 | | |
|  |  |  |  |  | H_2_S (µM) | | | | 42.2 | | | 0.8 | | | | 1.1 | | | 488.6 | | |
|  |  |  |  |  | Fe^2+^ (µM) | | | | n.d. | | | n.d. | | | | 172.1 | | | n.d. | | |
|  |  |  |  |  | Mn^2+^ (µM) | | | | n.d. | | | 337.8 | | | | 7.3 | | | n.d. | | |
|  |  |  |  |  | DIC (mM) | | | | 2.67 | | | 0.92 | | | | 5.71 | | | 5.70 | | |
|  |  |  |  |  | **δ**^13^C-DIC (‰) | | | | 6651 | | | 16963 | | | | 4215 | | | 5849 | | |
| Solid phase | | | | | TOC (%) | | | | 2.56 | | | 2.88 | | | | 3.21 | | | 2.92 | | |
|  |  |  |  |  | **δ**^13^C-TOC | | | | -14.6 | | | 704.3 | | | | -14 | | | -11.6 | | |
|  |  |  |  |  | TC (%) | | | | 2.24 | | | 2.68 | | | | 3.03 | | | 2.98 | | |
|  |  |  |  |  | **δ**^13^C-TC | | | | -14.3 | | | 3442.4 | | | | -12.1 | | | -11.1 | | |
| **B** | | | | |  | | | |  | | |  | | | |  | | |  | | |
|  | CH_4_ | + | 4 MnO_2_ | | | + | 7 H^+^ | | | 🡪 | HCO_3_^-^ | | + | 4 Mn^2+^ | | | + | 5 H_2_O | |  |  |
|  | CH_4_ | + | 8 FeOOH | | | + | 15 H^+^ | | | 🡪 | HCO_3_^-^ | | + | 8 Fe^2+^ | | | + | 13 H_2_O | |  |  |
|  | CH_4_ | + | SO_4_^2-^ | | | + | H^+^ | | | 🡪 | HCO_3_^-^ | | + | H_2_S | | | + | H_2_O | |  |  |

**Supplementary Tables 2 to 21.** Tables are accessible in a single spreadsheet under: <https://doi.org/10.5281/zenodo.14055788> )

**Supplementary Figure 1.** Microbial community composition at the end of the long-term incubations, based on relative abundances of 16S rRNA gene amplicon sequencing variants (ASVs). **A** Major phyla, **B** Archaea, **C** Bacteria**, D** ‘*Ca.* Methanoperedens’ genus ASVs, **E** Aerobic methane oxidizing bacteria (MOB) and, **F** Potential sulfate-reducing bacteria (SRB).

**
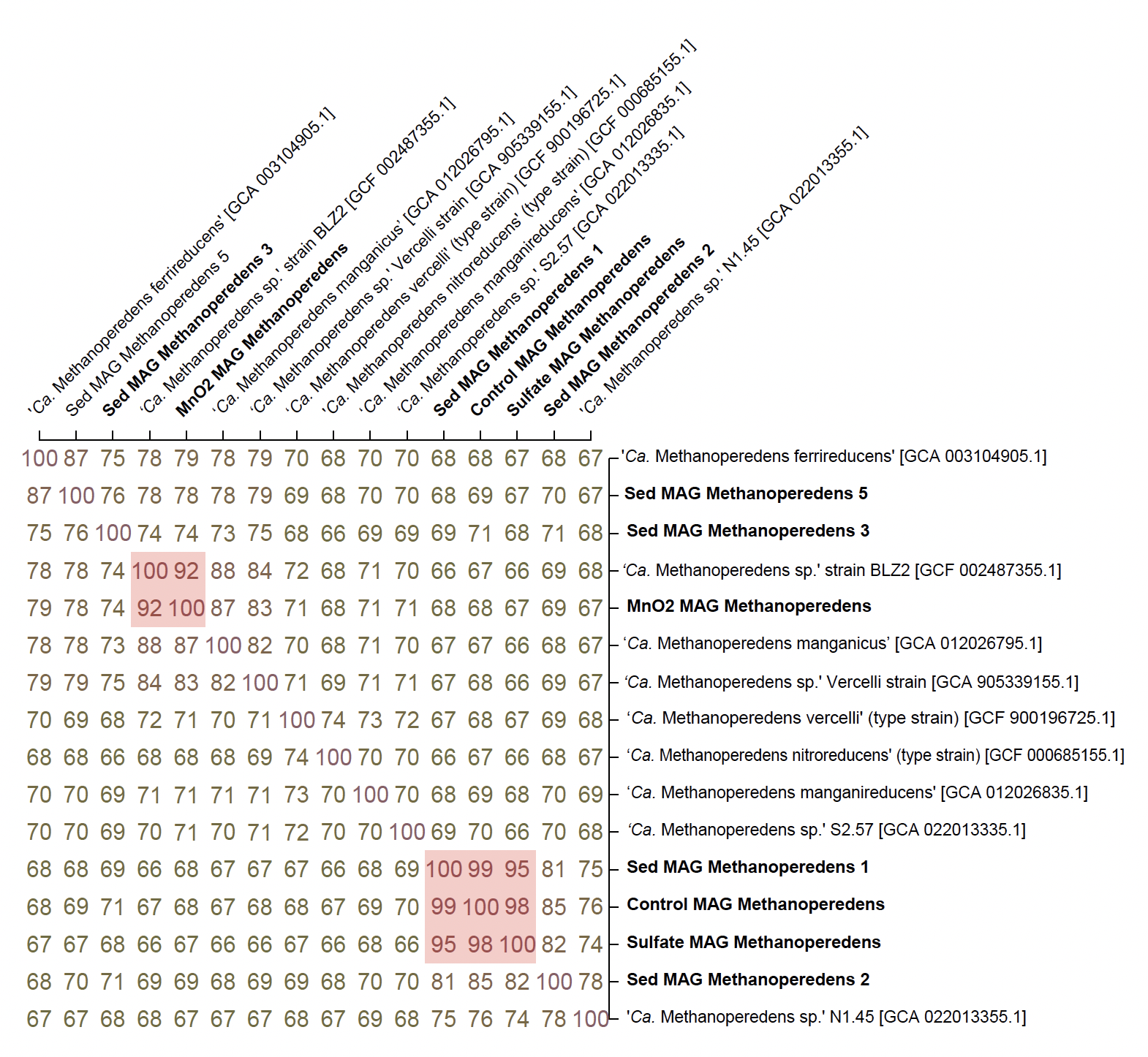
**

**Supplementary Figure 2.** Average amino acid identity (AAI) matrix built with the current Lake Cadagno ‘*Ca.* Methanoperedens’ spp. MAGs as well as MAGs belonging to reference bioreactor ‘*Ca.* Methanoperedens’ spp. enrichments. MAGs that are considered same species are highlighted in red (≥90% AAI) (Konstantinidis *et al.*, 2022). MAGs are labelled with the given NCBI name id (biosample reference) and identifier in squared brackets.

**Supplementary Figure 3.** Multi-heme *c*-type cytochromes (MHC) family domain tree including the current study’s ‘*Ca.* Methanoperedens’ MAGs and the reference ‘*Ca.* Methanoperedens’ bioreactor enrichments: *'Ca.*Methanoperedens ferrireducens’ (Fe-AOM) (ferri), *'Ca.*Methanoperedens manganicus (Mn-1)’ and *'Ca.*Methanoperedens manganireducens (Mn-2)’ (Mn-AOM), *'Ca.*Methanoperedens nitroreducens’ Type Strain (retentate) (electrode/nitrate/iron-AOM), *'Ca.*Methanoperedens Vercelli’ (electrode-AOM) (Methanoperedens). In the legend, from left to right: MAGs labelled based on current study origin (with total MHC domain counts in parenthesis), reference MAGs colored based on electron acceptor amendment (manganese oxides, iron oxides, multiple or electrode) as well as MHC expression (either high or medium) of the reference MAG proteins and proteins classified as nanowires (OmcZ). Branch lengths indicate the average number of amino acid substitutions per site. Black dots at nodes indicate robust branching with bootstrap values > 70%.


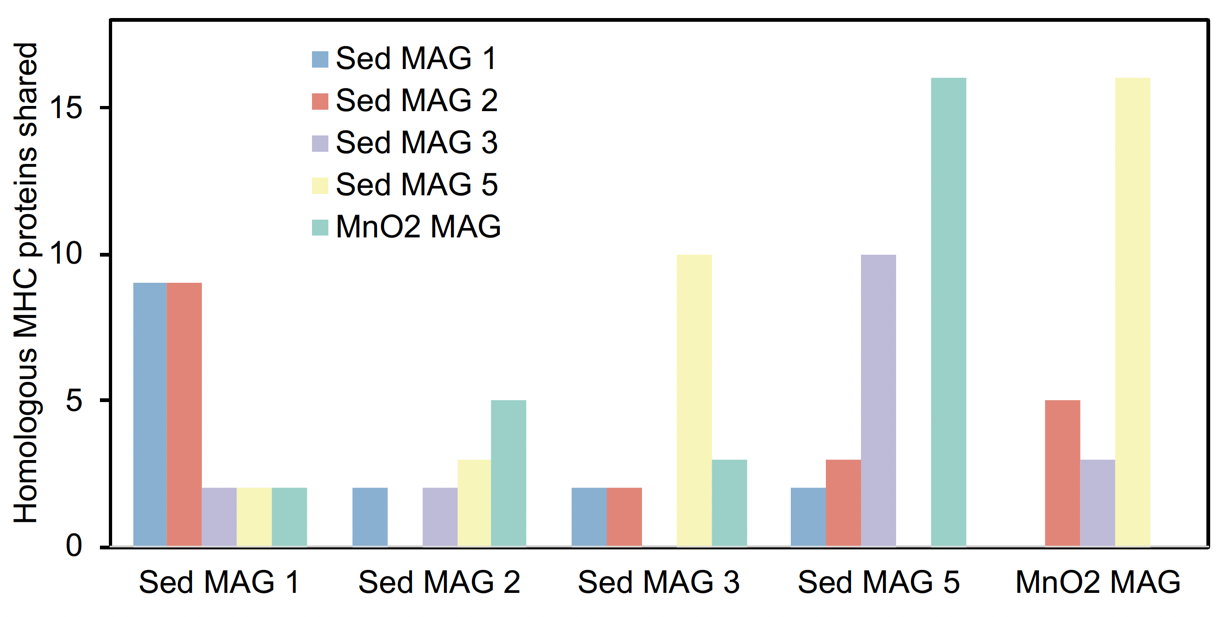


**Supplementary Figure 4.** Multi-heme *c*-type cytochromes (MHC) family domain similarity among the Lake Cadagno ‘*Ca.* Methanoperedens’ MAGs. Considered as homologous protein when the amino acid similarity was >70% of the MHC domain.


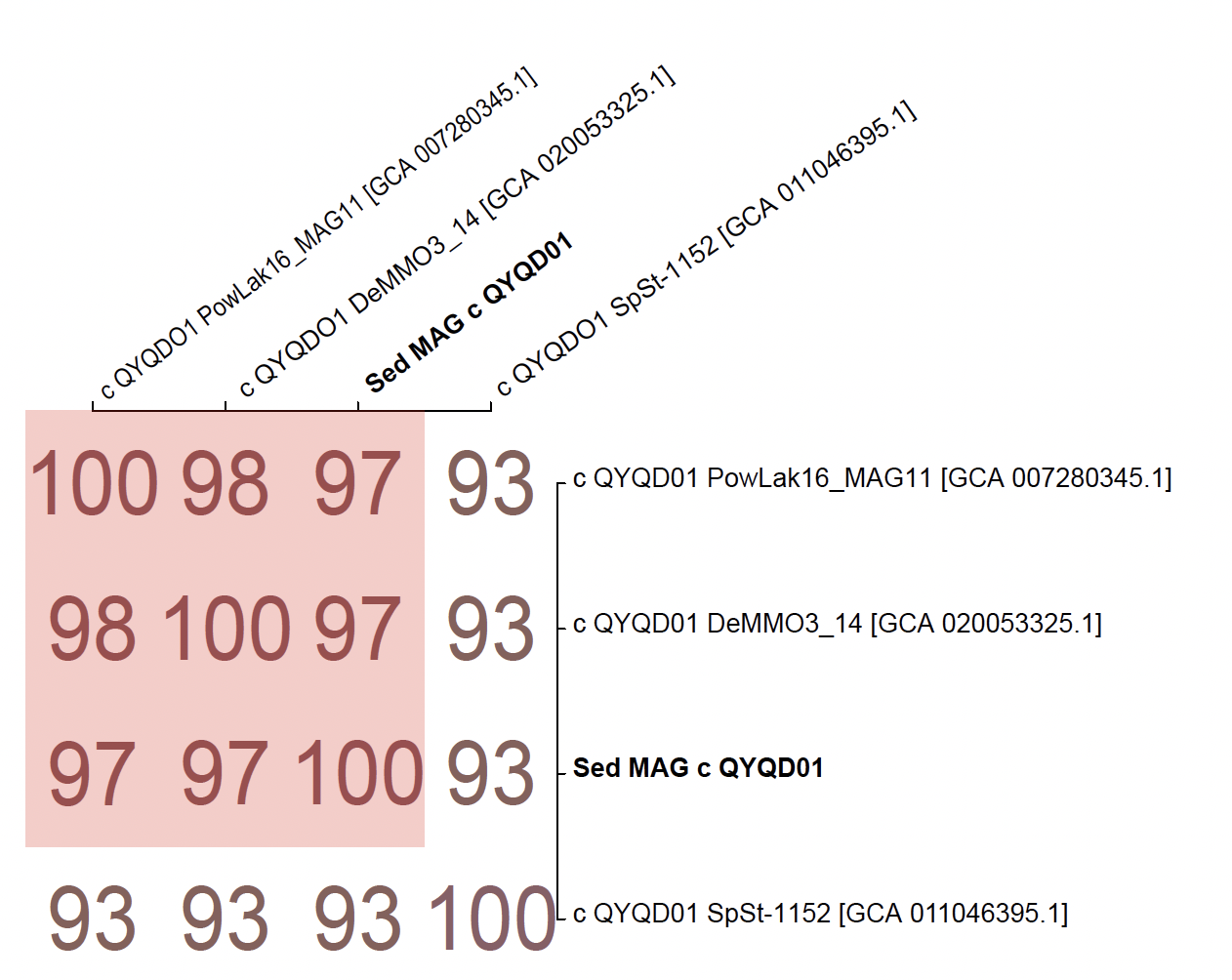


**Supplementary Figure 5.** Average nucleotide identity (ANI) matrix built with current study’s *Desulfobacterota* class *QYQD01* and high-quality reference MAGs (>80% complete) downloaded from GTDB. MAGs that are considered same species are highlighted in red (≥95% ANI) (Jain *et al.*, 2018). MAGs are labelled with the given NCBI sample/isolate name and the GenBank assembly identifier in squared brackets.

**
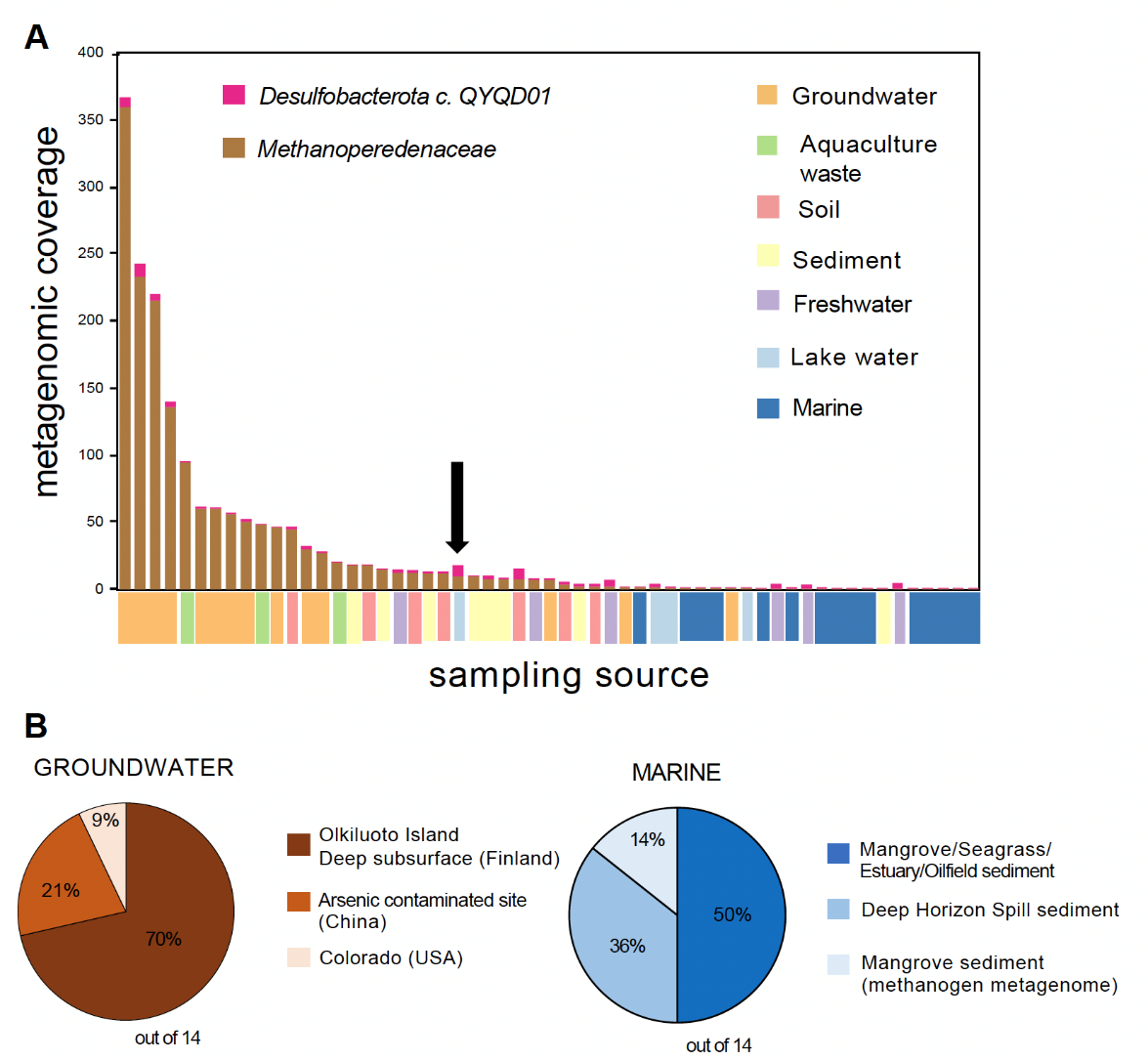
**

**Supplementary Figure 6. A** Environmental distribution of co-occurring *Methanoperedenaceae* and *Desulfobacterota* class *QYQD01.* Shown are Sandpiper (via SingleM)-based SRA search results as abundance plots for *Methanoperedenaceae* and *Desulfobacterota* class *QYQD01*, based on metagenomic coverage (y-axis) across different metagenomic studies (x-axis). Note that the ‘Others’ category in the pie-chart (not plotted in x-axis) harbors sites such as the iron-rich Deep Mine Microbial Observatory (DeMMO) from Casar *et al.* 2021. Metagenomic studies are arranged from left to right based on metagenomic coverage, and color-coded according to their source environment. The arrow indicates the Powell Lake (PowLake) metagenome where both metagenome assembled genomes (MAG) from ‘*Ca.* Methanoperedens’ and *Desulfobacterota* class *QYQD01* were retrieved **B** Proportions (%) of metagenomes from groundwater and marine samples, respectively, harboring both *Methanoperedenaceae* and *Desulfobacterota* class *QYQD01*.
